# Eye Movements to Natural Images as a Function of Sex and Personality

**DOI:** 10.1371/journal.pone.0047870

**Published:** 2012-11-30

**Authors:** Felix Joseph Mercer Moss, Roland Baddeley, Nishan Canagarajah

**Affiliations:** 1 Department of Computer Science, University of Bristol, Bristol, Avon, United Kingdom; 2 Department of Experimental Psychology, University of Bristol, Avon, United Kingdom; 3 Department of Computer Science, University of Bristol, Bristol, Avon, United Kingdom,; CSIC-Univ Miguel Hernandez, Spain

## Abstract

Women and men are different. As humans are highly visual animals, these differences should be reflected in the pattern of eye movements they make when interacting with the world. We examined fixation distributions of 52 women and men while viewing 80 natural images and found systematic differences in their spatial and temporal characteristics. The most striking of these was that women looked away and usually below many objects of interest, particularly when rating images in terms of their potency. We also found reliable differences correlated with the images' semantic content, the observers' personality, and how the images were semantically evaluated. Information theoretic techniques showed that many of these differences increased with viewing time. These effects were not small: the fixations to a single action or romance film image allow the classification of the sex of an observer with 64% accuracy. While men and women may live in the same environment, what they see in this environment is reliably different. Our findings have important implications for both past and future eye movement research while confirming the significant role individual differences play in visual attention.

## Introduction

Folk psychology has always been generous in affording differences to women and men. Research has found support for many of the gender stereotypes: the aggressive man and anxious woman schemas are evidentially sound [1]; women are more sensitive to social cues [2]; and boys engage in more risky behavior [3]. Such behavioural differences may, in part, be attributed to sex hormones affecting cerebral organization early in life, resulting in significant anatomical differences between the brains of men and women [4]. These include sex differences in the neurophysiological systems associated with anxiety [5] and reward [6], [7]. A person's sex is therefore an important factor that influences many of the decisions people make.

One decision, made by humans three times every second, is where to look. Despite primates being highly visual animals, their foveated vision delivers high-resolution visual information from only about two degrees of the visual array. The decision where to fixate is, therefore, not only very frequent but also very important. Locating the most rewarding and behaviorally-relevant stimuli is a difficult problem. Understanding the nature of the top-down and bottom-up factors that combine to determine where an individual will fixate is still a significant challenge.

Perhaps surprisingly, only a limited amount of work has focused upon the impact of observer sex on eye movements: women have been shown to be more sensitive to social gaze cues than men [8], while men fixate more on the nose region when recognizing emotion [9]. Sexual imagery has also been shown to consistently induce fixation patterns congruent with the sexual motivations of the viewer [10]. Furthermore, the analysis of fixation behavior with respect to the enduring characteristics of the observer is markedly underdeveloped. Personality has been shown to affect where people look while viewing fearful faces, with neuroticism predicting time spent fixating eye regions [11]. Optimists have also been shown to display increased vigilance towards positive image regions, while pessimists show corresponding biases towards negative imagery [12]. Although these studies highlight the influence individual differences can have on where people look, they do so over a very limited range of stimuli.

Here, we recorded the eye movements of 52 observers whilst they evaluated three different dimensions of the meaning of 80 different images with a wide range of content. Using information theoretic and Bayesian techniques, we attempted to answer the following questions: (1) are there differences between how men and women view the world; (2) what are these differences; (3) how do they vary with viewing time, image semantics and the viewers' task and personality; and (4) why do we observe these differences?

## Methods

### Ethics Statement

The protocol followed for data collection and analysis described in the current study was approved by the University of Bristol Faculty of Science Human Research Ethics Committee. Written and informed consent was obtained from each participant.

### Participants

Fifty-two individuals participated (26 women, 26 men) with age ranging from 19 to 47.

### Stimuli & Apparatus

Eighty stimulus images were chosen from a larger set of 260 (16 stills from action films, 16 stills from romance films, 16 stills from wildlife documentaries, 16 surrealist and 16 non-surrealist art pieces). The final 80 images were chosen to maximize semantic variation (see File S1 for more details). Image aspect ratios were locked before scaling them up or down to achieve maximum screen coverage. The background was filled with black.

A Tobii ×50 eye-tracker was used to record the gaze data at 50 hz. A fixation was defined as any interval in which gaze remained within 0.5 degrees for 80 ms or more. The eye-tracker was paired with a 17-inch CRT display at a resolution of 768×1024. The experiment was coded using MATLAB with the psychophysics [13] and talk2tobii [14] toolboxes. To record participants' image evaluations a custom-modified joystick was used with the handle extended to 80 cm. A fixed chin rest kept participants' heads steady.

An online questionnaire recorded age, sex, and two personality inventories: the 100-item IPIP representation [15] of the Five Factor Model [16] and the 45-item UPPS impulsivity scale [17].

### Procedure

Participants sat 60 cm from the display and viewed three blocks of trials that differed only in the task they were assigned. For each block, participants used the lever to rate each of the 80 images in terms of how much they liked the image (Evaluation), how stimulating they found it (Potency) or the amount of movement it contained (Activity). These three dimensions were chosen as they correspond to Osgood's semantic differential [18]. Each task was explained by showing the participant a list of words or phrases they might associate with the extremes of each dimension, these can be seen in Table 1. Each trial began with a fixation cross displayed in the centre of the screen for 400 ms, followed by a blank grey screen for a further 100 ms. An image was then presented for 5 seconds, during which participants were asked to evaluate the image using the lever with their right hand. After 5 seconds the image disappeared and the participant was presented with an onscreen cue to return the lever to the “neutral” position before the next trial began.

**Table 1:** Words associated with the each extreme of the three dimensions of Osgood's semantic differential.

Dimension	Positive	Negative
Evaluation	Nice, Beautiful, Lovely	Horrible, Awful, Ugly
Potency	Strong, Arousing, Impact, Reactionary	Weak, Boring, Pedestrian
Activity	Action, Speed, Movement	Passive, Calmness, Relaxed

Participants rated each of the 80 images with respect to these three axis. Before a block (corresponding to one of the three dimensions), they were shown these words as examples of what they might want to be looking for in the images.

Before each block, participants were given a clear onscreen definition, describing the criteria by which they were expected to rate the following images. Participants were then given 5 practice trials from a separate set of images, receiving feedback regarding their choice after each. After calibration had been completed (and an error of below 0.5 degrees had been achieved), participants were free to start viewing the trials in that block. Participants, therefore, viewed each image three times estimating its evaluation, potency and activity. Both the order of blocks and the presentation of images within each block were randomized. On completion of the three blocks, participants were directed to the online questionnaire.

### Analysis

The majority of the analysis was performed after transforming different sets of fixations into probability density functions (PDFs). This was achieved by, first, binning the number of fixations at each pixel, before smoothing the resulting two-dimensional distribution using a Gaussian kernel (with a standard deviation of 0.85 degrees). These fixation maps were then transformed to PDFs by normalizing so that they summed to one. We used two forms of PDFs. The first simply took the fixation locations and calculated the density. The second weighted each fixation by its temporal duration, and in doing so representing spatio-temporal fixation density rather than simply spatial fixation density.

**Figure 1:**
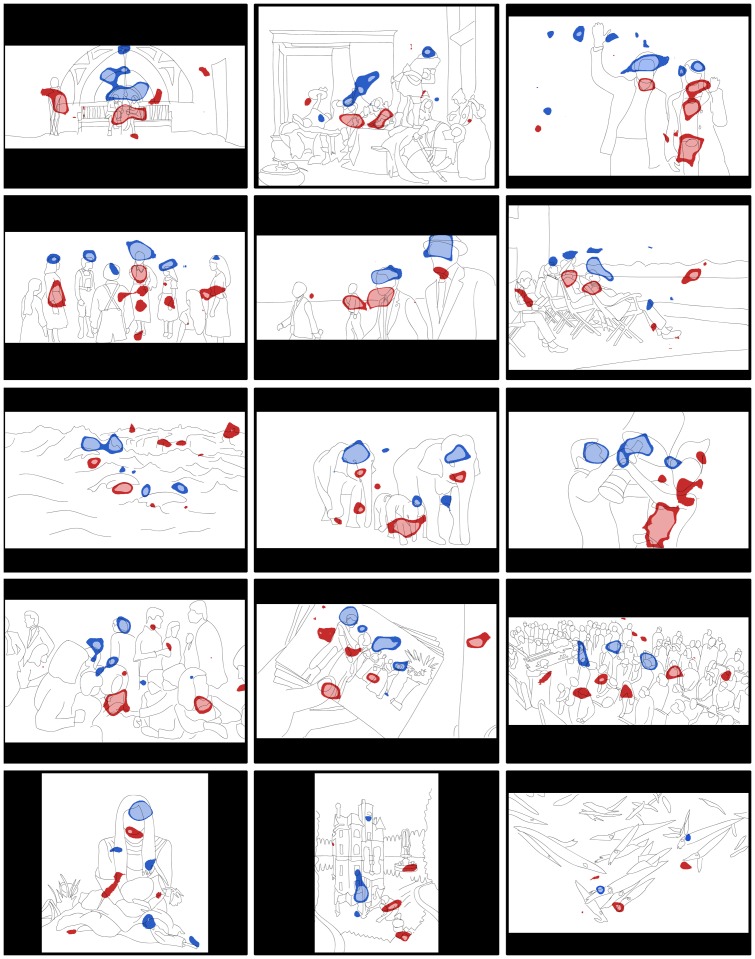
Images that produced the most distinct eye movements largely depicted social scenes. Significant differences (blue = men; women = red; dark = p<.05; light = p<.01) displayed for the top fifteen images that produced the most discriminating eye movements and the image that produced the least (bottom right). These images (displayed in full color during the experiment) largely depicted social scenes.

Male and female PDFs, created in this way, were then subtracted from one another to form difference images illustrating regions favored by the two respective groups (see Figure 1). To identify which regions were significantly different, 200 such difference images were generated, each time made by resampling original data with replacement (the difference images were bootstrapped). A Z-test was then used to test if each pixel in the difference image was reliably different from zero.

Temporal variation was explored by analysing how the spread of fixations developed from the beginning to the end of a 5 second trial. For each image, a chronological series of PDFs was calculated from sets of the first to the fifteenth fixations. The spread of each of these 15 distributions was quantified by calculating their entropy. The entropy of male and female PDFs were subtracted from the total entropy then compared to one another. This metric, therefore, measures the Shannon information each PDF provides about being either male or female. Male and female information was calculated for each fixation and each image. Mean estimates with standard errors were then calculated for the information provided by each sequential fixation during 5 seconds of viewing.

The sex of each participant was predicted by calculating whether it was more probable their fixations originated from either the male or the female PDFs, created using the fixations from the remaining 51 participants. The likelihood that a set of test fixations was female (or male) was taken as the product of the probabilities of each of those fixations coming from the female (or male) PDF. To turn these into (posterior) probabilities, these likelihoods were normalized by dividing them by the sum of the likelihood of being male and the likelihood of being female. This classifier is, therefore, the naïve Bayes classifier (naïve since it ignores the correlations between the likelihoods of different fixations), assuming a 50% prior for sex (which was correct for this experiment). This process is expressed formally in Equation S1.

As female observers were just as likely as male observers, the prior terms that should normally weight each likelihood cancel out. Eighty classifiers were created this way using the data from each of the stimulus images. Each classifier returned a probability of each participant being correctly classified as either male or female. In Bayesian terms, this value is equivalent to the posterior probability of a given participant being correctly classified as a man or woman based upon their fixations while viewing a given image, from this point on, however, it will be referred to as classification accuracy. These data were then subsequently used to explore which kinds of images and personalities were most likely to lead to correct classification of the viewers' sex, based on their fixation behavior.

Ten-fold, cross-validated logistic regression models were trained to predict these accuracy scores from the personality data. The significance of the beta values was evaluated by bootstrapping the data 200 times (sampling with replacement) before using Z-tests to indicate whether each beta value reliably fell either side of zero. Bonferroni correction was used to correct for multiple comparisons.

## Results

Independent samples t-tests showed male fixation durations (M = 305 ms, SD = 230 ms) to be reliably shorter than female fixation durations (M = 320 ms SD = 250 ms), *t*(179797) = 9.79, *p*<.001,) while female saccade amplitudes were significantly larger (M = 4.39 degrees, SD = 3.93 degrees) than male saccade amplitudes (M = 4.23 degrees, SD = 3.8 degrees; *t*(179797) = 9.09, *p*<.001).

**Figure 2:**
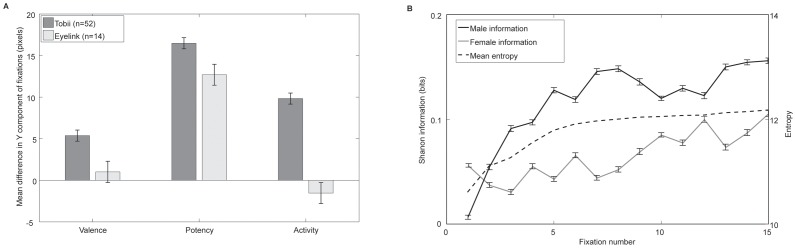
Women consistently fixated lower than men while there fixation distributions were more spread out than those of men. Panel A illustrates how the mean Y component of female fixations were lower than their male counterparts, especially during the potency block. This effect was replicated using a different, more accurate eye tracker and different participants. Panel B shows entropy calculations of the fixation maps show how, as expected, entropy increased with fixation number. Men's fixation distributions contained higher information than women's indicating women were employing more exploratory and diverse visual strategies, especially around the seventh fixation. Error bars are the standard error of the mean.

Entropy of both male and female fixation plots increased from the first to the seventh fixations: over time the spread of the distribution of fixations widened. The difference between the entropy of the male or female distributions individually, and the entropy of the averaged distribution measures the amount of information provided by the fixation distribution of a given sex: the information given by the female fixation distributions increased faster and to a higher level than their male counterparts (see Figure 2B).

**Figure 3:**
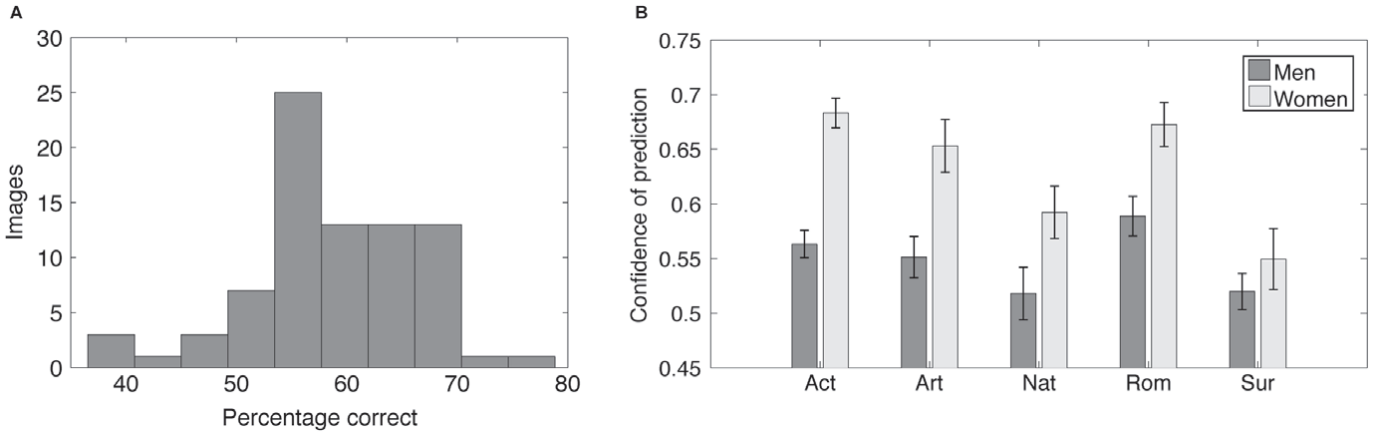
Sex classification accuracies spanned from 40% to almost 80% while fixations from different image categories produced significantly different levels of performance. Panel A displays the distribution of accuracies. Panel B shows which image categories produced the most discriminable fixations. Women, in particular produced more predictable fixations when viewing images that typically contained people. Error bars are the standard error of the mean.

Classification of the data from the 80 images produced accuracies that reached 79% with a mean of 59%. The distribution of classification accuracies can be seen in Figure 3A. Independent samples t-tests on the model accuracies indicated that women were classified significantly more accurately than men (*t*(4158) = 6.49, *p*<.001) and that weighting the PDFs by duration (the amount of time at given locations rather than simply the number of fixations) significantly improved classification of women, *t*(4158) = 3.1, *p* = .002, but not men *t*(4158) = 0.62, *p* = .53. Mean classification increased a single percentage point to 60%. All subsequent analyses were, therefore, carried out on duration-weighted fixation distributions.

While the highest performance was observed using the data from all three tasks, there was a significant difference to be found between classification accuracies of individual tasks *F*(2) = 4.545, *p* = .012. Post-hoc analyses using Tukey's Honestly Significant Difference (HSD) indicated the only significant difference (*p* = .008) was between data from the potency task (mean 58%) and data from the evaluation task (mean 54%). Differences in accuracy between data from the activity task (mean 56%) and evaluation task (*p* = .254) and potency task (*p* = .328) were not significant.

Figure 3B illustrates correct classification varied significantly with image class (*F*(4) = 9.105, *p*<.001). Post-hoc analyses (all *p*<.001) using Tukey's HSD revealed romance and action image accuracy (mean 64%) to be significantly higher than those of the nature and surrealist images (55%), while non-surrealist art images (62%) yielded significantly higher mean accuracies than surrealist art images (54%).

Whilst the semantic class of the image was related to classification accuracy, the raw meaning (as measured by the average semantic differential scores) was only marginally significant (all *p*>0.05).

Despite the raw meaning of an image being only loosely related to the probability of correct classification, the relative meaning (average female ratings subtracted from male ratings) yielded more significant correlations. The extent to which women rated an image more positive than men (*r*(78) = .414, *p*<.001), more potent than men (*r*(78) = .327, p = .003), and the absolute difference in activity (*r*(78) = .266, *p*<.017) were all significantly correlated with the classification accuracy.

**Figure 4:**
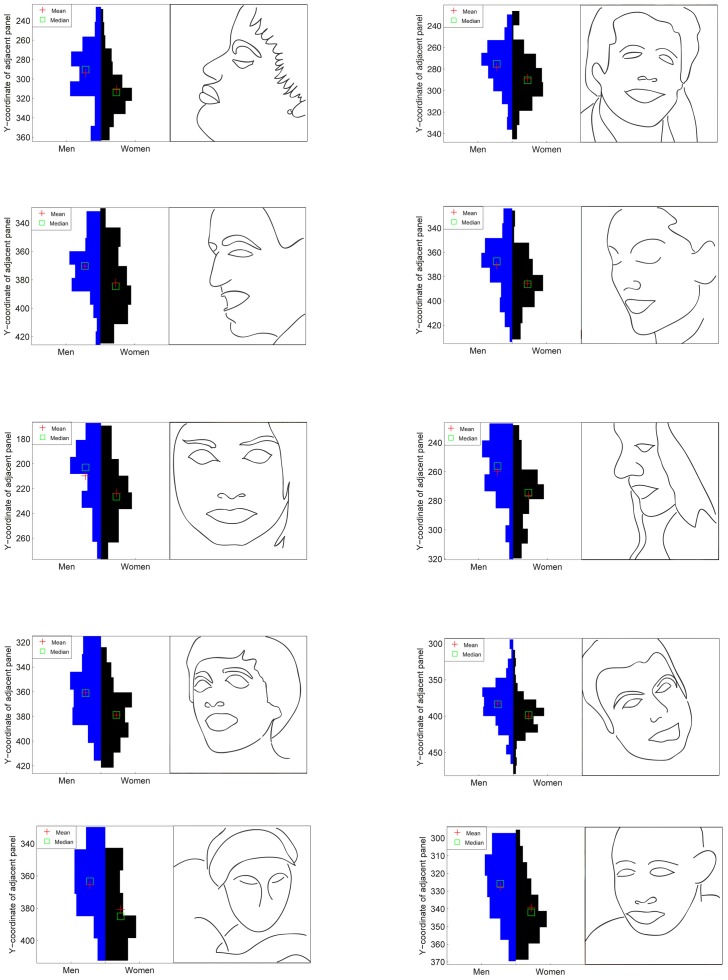
Particularly while viewing images depicting people, women looked marginally below salient features. Violin plots illustrate how the difference in the distribution of Y-component fixations when fixating faces is likely to be behaviorally significant. While the male distributions tend to center on the eyes of the faces, the distribution of female fixations are shifted down to the nose or even the mouth.

A mean vertical difference of 10.5 pixels between male and female fixations was found to be highly significant *t*(179797) = 19.35, *p* = <.001. The magnitude of this effect varied according to task, with the potency condition eliciting the largest effect, *t*(59301) = 17.47, *p*<.001, then activity, *t*(59871) = 10.46, *p*<.001 and finally evaluation, *t*(60621) = 5.68, *p*<.001. Cross-correlations of the male and female PDFs revealed a vertical shift in the image PDFs ranging from 0 to 17 pixels. The effect was correlated with prediction accuracy suggesting that the effect was contributing significantly to classification, r(79) = 0.51, p<.001. The vertical offset was greatest when viewing people-based images (romance, action or non-surrealist art, *t*(78) = 2.41, *p* = .018) and when estimating potency *F*(2) = 70.8, *p*<.001. Figure 4 illustrates the effect when the target is a face. The effect was replicated using a different eye tracker (Eyelink 2000) and 14 new participants (see Figure 2A). Again, women looked significantly lower than men overall, *t*(48850) = 3.87, *p*<.001, and particularly in the potency condition, *t*(16186) = 7.05, *p*<.001 (evaluation, *t*(16571) = 0.55, *p* = .55, and activity *t*(16089) = 0.84, *p* = .40, were not significant).

**Figure 5:**
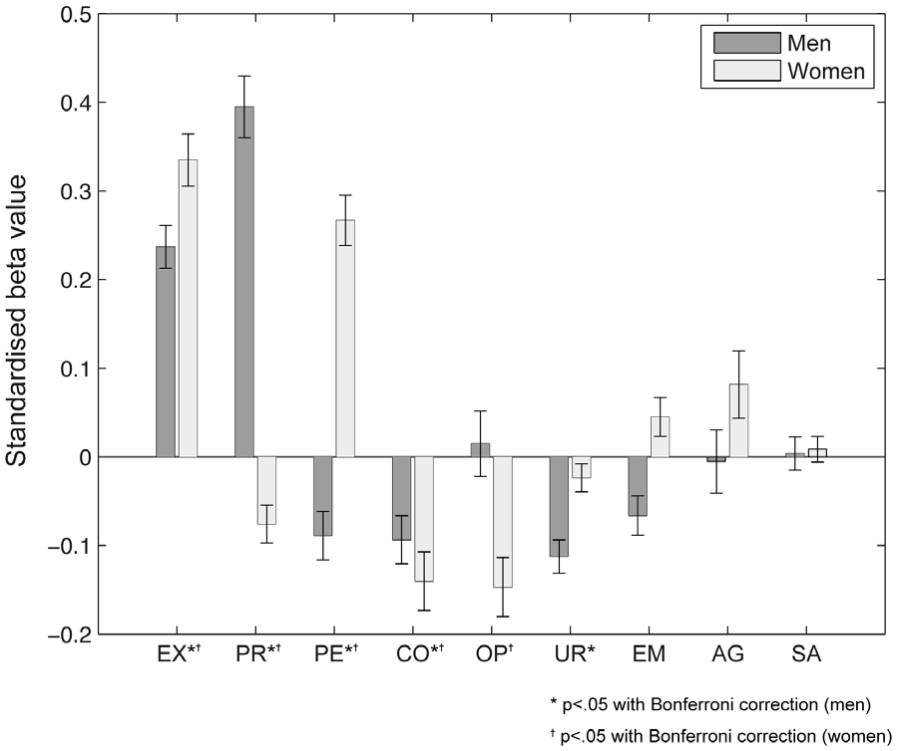
Personality predicts the accuracy of fixation-based sex classification. Standardized beta values of a logistic regression model trained with personality data to predict sex classification accuracy. Positive beta values represent traits that are likely to be seen in correctly classified individuals while negative betas indicate traits prevalent in misclassified participants. After Bonferroni correction, extraversion (EX), premeditation (PR), perseverance (PE) and conscientiousness (CO) were still significant for both men and women. Openness to experience (OP) was also left significant for women and urgency (UR) for men. Emotional stability (EM), agreeableness (AG) and sensation-seeking (SE) were not significant for either men or women. Error bars represent the standard deviation of the 200 bootstrap estimates.

Logistic regression models trained with personality data to predict accuracies yielded predictions that correlated significantly with the real values, *r*(414) = .358, *p*<.001. The standardized beta values for this model can be seen in Figure 5. After Bonferroni correction, constructs that significantly predicted female accuracies were extraversion (**β** = 0.334) perseverance (**β** = 0.264), openness to experience (**β** = −0.1476), conscientiousness (**β** = −0.139), and premeditation (**β** = −0.078). Constructs that significantly predicted male accuracies after Bonferroni correction were perseverance (**β** = 0.395), extraversion (**β** = 0.236), perseverance (**β** = −0.091), conscientiousness (**β** = −0.092) and urgency (**β** = 0.114). Extraversion, in particular, influenced predictability in women, *r*(24) = .47, *p* = .016.

## Discussion

We asked participants to rate 80 images on the three dimensions of the semantic differential: how pleasant (evaluation), how intense (potency), and how active (activity) the images were. The majority of fixations when performing these tasks were made to about 1–5 ‘hot spots’. Usually, the most informative regions of a scene are locations with people in them, and the most informative location of a person is generally their face (and in particular the region around the eyes). Unsurprisingly, therefore, the majority of these hot spots tended to be focused on people's faces (and particularly their eyes). The second most common location for a hot spot was to non-eye locations on people. The rest of the fixations were more evenly spread out to a number of more ‘exploratory’ regions.

This pattern was true for both men and women, for the three tasks, for the different classes of image, and for the people with different personalities. Despite this, there were numerous robust differences in the fixation distributions between men and women, mainly in the relative proportions of eye movements made to eyes, non-eye location in people, and exploratory locations.

Women, on average, tended to be more exploratory, making more fixations to non-face locations. This observation was mirrored both by the fact that men's eye movements were 4% shorter but 4% more frequent than women's, and by the entropy-based measures where the female fixation distributions were more spread out (and continued to spread out for longer). This exploratory behavior produced more distinctive female fixation maps, partly explaining why women were more reliably classified than men: if an individual made exploratory eye movements, they were more likely to be classified correctly as female.

A second difference was that men and women find different things interesting, and this being reflected in their eye movements. The classification accuracy was correlated with the difference in how the male and female participants evaluated individual images. Images that women, compared to men, rated as more positive, more potent, and more different with respect to activity were reliably more accurately classified. Together, these effects explained a quarter of the variance in classification accuracy.

One example of this difference in interests can be seen in the difference in fixations to heterosexual couples. All participants preferentially fixated female figures, but this effect was more pronounced in women (61% to female figures; 39% to male figures) than in men (53% to female figures; 47% to male figures). Inspection of the difference maps (two of which are illustrated in Figure 1) indicated that this difference was largely due to women scanning more of the entire female figure while men generally concentrated fixations on the face. Action, romance and non-surrealist art images formed a set of people-based images that produced significantly higher classification accuracies than nature-based and surrealist art stimuli. In addition to the interpretive scope afforded to the social scenes, action and romance films are, to a certain extent, shot to engage male and female audiences respectively; therefore, it is perhaps not surprising that stimuli taken from these genres produced the most discriminatory fixations.

The differences between viewing in men and women were also moderated by task. In particular, classification accuracy was largest when the task was to rate the potency of the image. The largest difference was between the potency and evaluation tasks: potency induced more discriminating fixations than evaluation. An explanation lies in the kind of responses an observer might anticipate when participating in these different tasks. In Table 1, the words that participants were encouraged to associate with the evaluation task were primarily aesthetic: ‘nice’ or ‘horrible’ and ‘beautiful’ or ‘ugly’. On the other hand, the words associated with the potency judgement carried more emotional weight: ‘strong’ or ‘weak’ and ‘impact’ or ‘boring’. An important difference between the search for these two types of information is that an aesthetic judgement carries less behavioral relevance than an emotional one: deciding a scene is superficially unpleasant may make for uncomfortable viewing, but it does not necessitate a swift change of behavior. However, a highly emotionally-charged scene, whether pleasant or not, is likely to dictate an important change in circumstance and a shift in behavior. The words to describe the potency axis, therefore, may have primed the participants to look for stimuli that necessitate a more active response than those in the evaluation task. While our data indicated the raw semantic differential content of an image had little effect upon fixation discrimination, it appears the anticipation of such content does have an effect. A recent meta-analytic study has documented how many studies have converged upon the conclusion that sex differences in the impulsivity trait are explained not by increased male sensitivity to reward but by increased female sensitivity to punishment [19]. One interpretation of the task-based differences seen here is that women were more inclined to anticipate a threat than men in the potency task and adjusted their visual strategies accordingly.

Another effect that varied similarly according to task was a function of the Y component of fixations. The basic effect can be seen in the Figure 2A and, more specifically, when the target is a face in Figure 4. As stated previously, the majority of fixations made by both men and women are to a small number of hot spots. The fixation distributions of female fixations to these hot spots are, in most situations, shifted away and predominantly below those made by men: relative to those made by men, women's eye movements appear to be repelled slightly from obvious locations of interest. This effect was strongest when viewing action and romance images and when searching for potency. Why?

One explanation appeals to physiological sex differences in the human eye: the lower, central and left subfields of the human retina have been found to be reliably thicker in men than in women [20]. By fixating slightly below a given target, light from the inverted target image would be projected onto a slightly lower (and for a woman thinner) part of the fovea. If this were to be a correct explanation, a similar leftward bias would also be expected. However, such a bias was not observed and, furthermore, the explanation does not account for the effect being moderated by both image content and observer task.

A similar behavioral effect has been observed in the face perception literature: a face is perceived to be more feminine if the gaze is averted downward [21]. The authors suggested looking down is an evolutionary adaptation to facilitate sex recognition by making the sexually distinct brow-lid distance more salient. Our data indicate the vertical shift is, indeed, heightened while viewing social scenes; however, the pattern of results observed here is both more subtle and intricate than might be expected if it was generated by a socially expressive gaze cue. More specifically, such an explanation does not account for why the effect was significantly more pronounced during the potency task.

An alternative theory appeals to the difference in threat perception between men and women discussed earlier. The information-rich hotspots that dominate the fixation maps seen here also contain high levels of reward for the observer. However, some of these regions also carry threat or risk of punishment. One image feature that carries risk, and therefore causes eye movements to be directed away from a location, is a light source [22], [23], probably to suppress the temporary blindness that would be associated with fixating it. Light sources generate a very similar pattern of fixations to the one we observe here. At a higher level, whilst faces are often associated with reward, at least in the United Kingdom, direct eye contact can be a potentially threatening cue. Asking someone if they are “looking at me” is less a request for information, and more a challenge to combat. Figure 4 demonstrates the behavioral significance of the effect when the target is a face: despite the aversion being only a fraction of a degree, the difference can be between fixating the eyes region and the nose or mouth. Such a difference can be framed as a trade-off decision between reward and risk: fixating the eyes carries the highest reward but also the highest risk; fixating the nose or mouth meanwhile brings less reward but also avoids the associated potential threat. As stated earlier, behavioral studies [19] indicate that women are more sensitive to punishment than men, resulting in more risky male behavior. Is there, however, any reason to believe this effect is manifested in visual attention?

Recent evidence [24] suggests that reinforcement learning plays a much more significant role in the selection of eye movements than previously anticipated. Moreover, the physiological properties of one of the central areas involved in both the generation of eye movements and reinforcement learning, the basal ganglia [25], is known to be sexually dimorphic [26], [27]. This area, at first approximation, contains two pathways: a direct (or “go”) pathway that facilitates eye movements, and an indirect (or “no go”) pathway that inhibits them [28], [29]. Importantly, recent evidence indicates estrogen, the main female sex hormone, selectively affects the dopamine D_2_ receptor utilized primarily by the “no go” pathway [7]. One explanation for the difference we observed is that women have a relatively more active “no go” pathway. Under this interpretation, the reinforcement learning mechanisms known to operate in the basal ganglia learn over time that faces are not only associated with potential reward, but also threat. In this proposal, the basal ganglia learns to label all potential fixation locations in terms of the rewards (direct and D_1_-based pathway) and risks (indirect and D_2_-based pathway) associated with them, and chooses the locations that maximize reward whilst minimizing risk. We know that estrogen moderates both the D_2_ receptor (that inhibits eye movements), and the salience of emotional displays of danger [30]. This interpretation is most consistent with the effect being largest both when viewing people-based images and when searching for potency.

The effects documented thus far do not operate in isolation. Individuals high in the extraversion trait were more likely to be correctly classified as either male or female, whereas high scores in the conscientiousness trait decreased the likelihood of a correct classification. Extraverts were more likely to engage with the highly predictive people-based images, and in doing so, increased the probability of forming different interpretations and consequently seek out different visual information. By contrast, the conscientiousness trait describes highly organized and focused individuals whose information gathering strategies are less likely to be influenced by their interpretation of an image. Two of the impulsivity sub-dimensions (premeditation and perseverance) significantly predicted positively for one sex and negatively for the other. Premeditation was found to be the strongest predictor of correct classification in men but a predictor of misclassification in women. Premeditative individuals put a high value on information and are, therefore, likely to make more fixations to the eye regions of faces. While most women tended to fixate marginally below the eyes, those who scored highly in the premeditation trait may have been drawn more to these information-rich regions and consequently misclassified as men. The perseverance construct was a strong predictor of correct classification in women yet incorrect classification in men. This trait may explain part of the difference in entropy between the fixations distributions between men and women. Highly perseverant women would be inclined to continue gathering visual information from new locations for the duration of any given trial, in the process forming wide, high entropy distributions. Highly perseverant men engaging in the same strategy, however, would have been misclassified as women. Here we have described only some of a sizeable number of effects and interactions between viewing behavior and the characteristics of the viewer, and these will be the subject of a later paper: the viewer's sex is an important determinant of fixation behavior, but it is not the only one.

In summary, men and women look at the world differently. Men make more but shorter eye movements; women are more exploratory and are interested in different things. For many hot spots, women's eye movements are systematically shifted away and below the most obviously informative location, and this is greatest when primed for threat.

The broad implications of sex–divergent gaze affect both future technological applications and methodological considerations. Eye movements are a potentially rich source for viewer information and the current findings lay important groundwork for possible future implementations of user profiling. Methodologically, laboratories based in engineering departments, (where participants are primarily male), will get systematically different results from those in psychology departments (where participants are primarily female). Previous work on eye movements has shown that both visual salience [31], [32] and task [33], [34] affect eye movement behavior. Here, we have shown that the characteristics of the viewer (sex, chiefly among them) must be added to this list. Since where we look helps construct what we see, the visual worlds experienced by women and men can, at times, be very different.

## Supporting Information

Equation S1 **Formal expression of the naïve Bayes classifier used to predict whether a set of test fixations was either from a male or female observer.** *M* denotes a male or female model (a PDF), *F* a given set of test fixations and *f_i_* the *i*th fixation of *F*.
(PDF)

File S1 **Describes the pilot study that was used to identify the set of stimuli used in the main study.**
(DOC)
