# Supplementary material for: Eye Movements to Natural Images as a Function of Sex and Personality
Source: PLoS One. 2012 Nov 30;7(11):e47870. doi: 10.1371/journal.pone.0047870 (PMC3511485; doi:10.1371/journal.pone.0047870)
Supplement: Equation S1 — Formal expression of the naïve Bayes classifier used to predict whether a set of test fixations was either from a male or female observer. M denotes a male or female model (a PDF), F a given set of test fixations and fi the ith fixation of F. (PDF) [file pone.0047870.s001.pdf]

$$P(M_{female} \mid F) = \frac{\prod_{i=1:n}^{n} P(f_i \mid M_{female})}{\prod_{i=1:n}^{n} P(f_i \mid M_{female}) + \prod_{i=1:n}^{n} P(f_i \mid M_{male})}$$
