# Supplementary material for: Eye Movements to Natural Images as a Function of Sex and Personality
Source: PLoS One. 2012 Nov 30;7(11):e47870. doi: 10.1371/journal.pone.0047870 (PMC3511485; doi:10.1371/journal.pone.0047870)
Supplement: File S1 — Describes the pilot study that was used to identify the set of stimuli used in the main study. (DOC) [file pone.0047870.s002.doc]

**SUPPORTING INFORMATION (S1)**

A separate pilot study was conducted to determine the eighty images used as visual stimuli for the main study. The aim of the pilot study was to acquire a set of images that maximized semantic variation. Semantics were formalized according to their image class (source media) and their position in three-dimensional semantic differential space [1].

**Ethics Statement**

# The protocol followed for data collection and analysis described in the current study was approved by the University of Bristol Faculty of Science Human Research Ethics Committee. Written and informed consent was obtained from each participant.

**Participants**

Five individuals took part in this experiment, three were male and two were female. Age ranged from 24 to 46 and they had normal or corrected-to-normal eyesight.

**Stimuli**

Two hundred and sixty images were chosen from 5 different image classes (40-60 from each class): action film stills; romance film stills; wildlife documentary stills; surrealist art pieces and non-surrealist art pieces. The art pieces were manually selected from art archives on the internet, while the stills were manually extracted as screen grabs from DVDs. The stills taken from wildlife documentaries were exclusively from Planet Earth (2006) or The Blue Planet (2005). Action films included: *Die Another Day* (2002), *Apocalypse Now: Redux* (1979), *Gomorrah* (2008), *The Warriors* (1979), *Transformers* (2007), *The Thin Red Line* (1999) and *Inside Man* (2006). Romance films included: *Love Actually* (2003), *Kate & Leopold* (2002), *Ghost* (1990), *The Sound of Music* (1965), *Breakfast at Tiffany’s* (1961) and *He’s Just Not That Into You* (2009).

A website was created that displayed each of the images above nine sliders. Each slider correlated with one of the three semantic differential scales: (1) evaluation - nice/awful, beautiful/ugly and clean/dirty; (2) potency - light/heavy, weak/strong and small/large; and (3) activity - slow/fast, passive/active and dull/sharp.

**Procedure**

Participants were directed to the web address where each of the 260 images was displayed in a random order. Participants were then instructed to rate each image according to the scales beneath the image.

**Data Analysis**

A factor analysis was performed on the resulting data (using principal components analysis and varimax rotation) and, as expected, the factor loadings showed three main components with the three groups of sliders each correlating to a different factor. The rotated component matrix indicated the first component to be evaluation, the second activity and the third potency.

The factor loadings of each image were then used to create a new dataset, around a third of the original size. The images were first split up into their image classes before taking the respective two highest and lowest scoring images, along each factor. This yielded a new dataset of 80 images composed of 16 images from each image class, with a broad range of semantic variability spanning each.

**References**

1. Osgood CE, Suci GJ, Tannenbaum PH (1957) The measurement of meaning. University of Illinois Press.
